# Single-cell RNA-seq of cultured human adipose-derived mesenchymal stem cells

**DOI:** 10.1038/sdata.2019.31

**Published:** 2019-02-26

**Authors:** Xuanyu Liu, Qinqin Xiang, Fen Xu, Jiuzuo Huang, Nanze Yu, Qixu Zhang, Xiao Long, Zhou Zhou

**Affiliations:** 1 State Key Laboratory of Cardiovascular Disease, Beijing Key Laboratory for Molecular Diagnostics of Cardiovascular Diseases, Center of Laboratory Medicine, Fuwai Hospital, National Center for Cardiovascular Diseases, Chinese Academy of Medical Sciences and Peking Union Medical College, Beijing 100037, China; 2 Division of Plastic Surgery, Peking Union Medical College Hospital, Beijing 100730, China; 3 Plastic Surgery Department, the University of Texas MD Anderson Cancer Center, Houston, TX 77030, USA

**Keywords:** Cell biology, Mesenchymal stem cells, RNA sequencing, Gene expression analysis

## Abstract

Adipose-derived mesenchymal stem cells (ADSCs) show considerable promise for clinical applications in regenerative medicine. We performed a large-scale single-cell transcriptomic sequencing of 24,358 cultured human ADSCs from three donors. We provide a high-quality dataset, which would be a valuable resource for dissecting the intrapopulation heterogeneity of cultured ADSCs as well as interrogating lineage priming patterns for any interested lineages at single-cell resolution.

## Background & Summary

Mesenchymal stem cells (MSCs) are multipotent adult stem cells capable of self-renewal and differentiation into mesodermal lineages such as osteocytes, adipocytes and chondrocytes, as well as endodermal and ectodermal lineages^1^. MSCs show considerable promise for clinical applications in cell therapy and regenerative medicine, especially because they are easily accessible and hold no ethical concerns, in contrast to embryonic stem cells. MSCs can be isolated from almost all adult tissues (e.g., adipose tissue, dental tissues and bone marrow) and solid organs (e.g., lungs, liver and spleen)^2^. Among these sources, adipose tissue is widely regarded as the ideal source for MSC isolation, as it has obvious advantages over other sources. First, subcutaneous adipose depots are ubiquitous and easily accessible in large quantities with a minimally invasive procedure, namely, liposuction. Second, adipose tissue usually contains far more MSCs than other sources contain. For example, MSCs constitute up to 3% of all cells in adipose tissue versus 0.01% in bone marrow^3,4^. After isolation, these so-called adipose-derived mesenchymal stem cells (ADSCs) can be expanded *in vitro* while maintaining their differentiation potential during culture. Recently, ADSCs have emerged as a promising class of multipotent stem cells and have been widely applied in tissue repair and regeneration, such as soft tissue reconstruction, cutaneous wound healing and cardiac regeneration^5,6^.

Cellular heterogeneity is a general feature of biological tissues and exists even within seemingly ‘homogeneous’ stem cell populations, which are influenced by extrinsic microenvironmental factors or intrinsic factors^7^. Abundant evidence has demonstrated that MSCs in culture are intrinsically heterogeneous in phenotypes and functions, as reviewed by Phinney^8^. However, the cell-to-cell variability in a cultured MSC population cannot be fully described by a handful of cell surface markers. The lack of a thorough understanding of the cellular heterogeneity of MSCs has hampered the development of an efficient and reproducible clinical application. For cultured ADSCs, an immunophenotypic characterization with a panel of 242 antibodies has been reported^9^. However, no study to date has dissected the heterogeneity of cultured ADSCs in a systematic manner.

While the transcriptomic differences among cells are completely masked when population-level RNA sequencing is used, single-cell RNA-seq has shown itself to be a powerful tool to comprehensively dissect cellular heterogeneity in an unbiased manner with no need for any prior knowledge of the cell population^7^. Recent technical advances have enabled the transcriptomes of tens of thousands of cells to be assayed at single-cell resolution in a single experiment^10^. It is thus of great interest for us to take this unprecedented opportunity to dissect the cellular heterogeneity of ADSCs with large-scale single-cell transcriptomic profiling.

Lineage priming, first proposed for hematopoietic stem cells, represents a cellular state in which stem cells before differentiation induction express, albeit at a low level, a subset of genes associated with the differentiation lineage to which they have potential to commit^11^. For MSCs, the patterns of lineage priming was reported in human and mouse bone marrow-derived MSCs by population-level RT-PCR analysis^12^. However, population-level analysis, which averages expression across a population of cells, cannot discriminate between a mixture of cells with varying degrees of lineage bias and a homogeneous set of multilineage-primed cells; this limitation highlights the significance of single-cell analysis in studying lineage priming^13^.

Here, we performed a large-scale single-cell transcriptomic sequencing of 24,370 cultured ADSCs. We provide a high-quality dataset, which would be a valuable resource for dissecting the intrapopulation heterogeneity as well as interrogating lineage priming patterns for any interested lineages at single-cell resolution.

## Methods

### Ethical approval

This study was approved by the ethics committee of the institutional review board at Fuwai Hospital and Peking Union Medical College Hospital. All procedures involving human participants were in accordance with the ethical standards of the research committee and its ethical standards. Informed consent was obtained from all participants.

### Isolation & culture of human ADSCs

**Figure 1:**
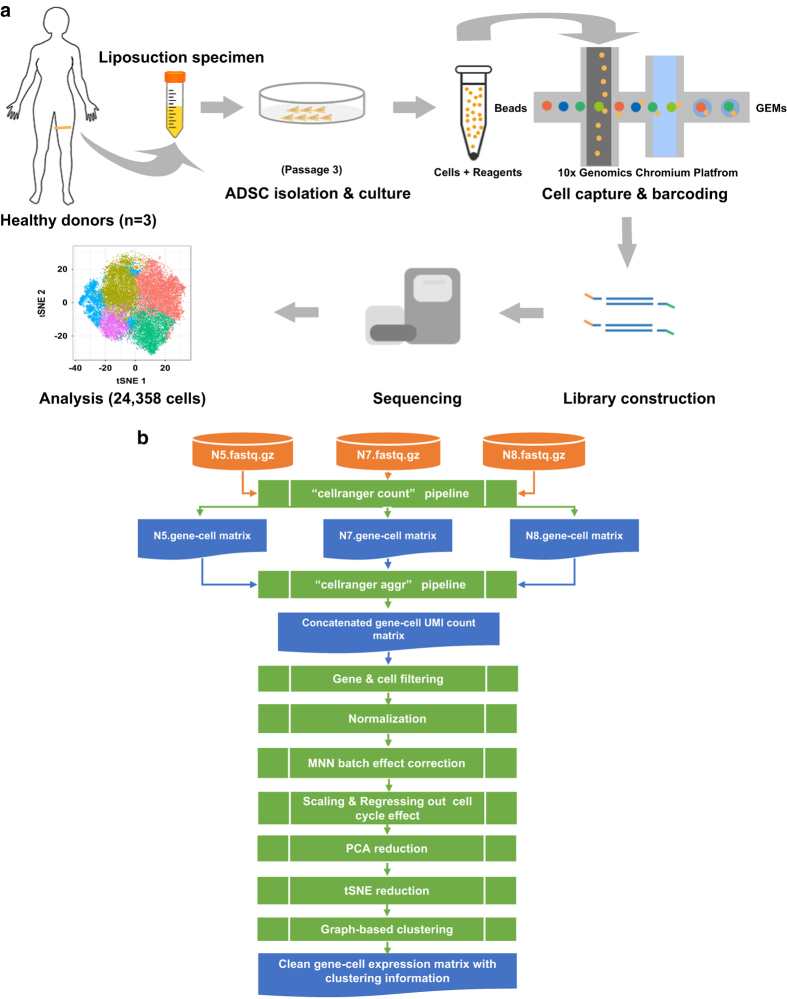
Overview of the experimental procedure. (**a**) Schematic representation of the experimental workflow. ADSCs were isolated from the liposuction specimens of three healthy, female donors. ADSCs that had been passaged three times were subjected to single-cell suspension preparation, library construction and sequencing. (**b**) Bioinformatic analysis workflow.

ADSCs were isolated from the liposuction specimens of three healthy, female donors (N5, N7 and N8) who underwent liposuction surgery for cosmetic purposes (Fig. 1a, Supplementary Table S1). The isolation procedure was performed as described previously^14^. Briefly, each liposuction specimen was washed with Hank’s balanced salt solution (HBSS) several times to eliminate blood cells. Then, it was digested with 0.1% collagenase supplied with 4% penicillin streptomycin solution (P/S) at 37 °C for 30 min. Subsequently, it was centrifuged at 1,500 rpm for 10 min. The pellets were resuspended in HBSS and filtered through a 100-μm strainer. The resulting cell suspension solutions were centrifuged at 1500 rpm for 10 min and resuspended in low-glucose Dulbecco’s Modified Eagle’s Medium (DMEM) with 15% fetal bovine serum (FBS) and 2% P/S to generate primary ADSC cultures.

### Preparation of a single cell suspension

ADSCs that had been passaged three times were used to prepare a single-cell suspension. Once at 50–60% confluence, the cells were digested with TrypLE™ Express (Thermo Fisher Scientific). Subsequently, the cells were centrifuged at 300 × g for 5 min, and the pellets were resuspended in HBSS with 0.04% BSA. The cell concentration was determined by Countstar (Aber Instruments Ltd). The target cell concentration (1 × 10^6^ cells per milliliter) was achieved by adding appropriate volumes of HBSS with 0.04% BSA. The cells were finally filtered using a 40-μm strainer to remove any cell debris or large clumps.

### Single-cell RNA-seq library preparation & sequencing

The 10x Genomics Chromium platform was used to capture and barcode the cells to generate single-cell Gel Beads-in-Emulsion (GEMs) by following the manufacturer’s protocol. Briefly, along with the reverse transcription master mix, cell suspensions were loaded onto 10x Genomics Single Cell 3′ Chips. During this step, cells were partitioned into the GEMs along with gel beads coated with oligonucleotides. These oligonucleotides enable mRNA capture inside the droplets by 30 bp oligo-dT after cell lysis and provide barcodes to index cells (14 bp) as well as transcripts (10 bp UMI). Following reverse transcription, cDNAs with both barcodes were amplified, and a library was constructed using the Single Cell 3′ Reagent Kit (v2 chemistry) for each sample. The resulting libraries were sequenced on an Illumina NovaSeq 6000 System in a 2 × 150 bp paired-end mode.

### Sample demultiplexing, barcode processing & UMI counting

Sample demultiplexing, barcode processing and UMI counting were performed by using the official 10x Genomics pipeline Cell Ranger v2.1.0 (https://support.10xgenomics.com). Briefly, raw base call files generated by Illumina sequencers were demultiplexed into reads in FASTQ format using the “cellranger mkfastq” pipeline (Data Citation 1). The raw reads were trimmed from the 3’ end to get the recommended number of cycles for read pairs (Read1: 26 bp; Read2: 98 bp). The reads of each library were then processed separately using the “cellranger count” pipeline to generate a gene-barcode matrix for each library. During this step, the reads were aligned to a human reference genome (version: hg19). Cell barcodes and UMIs associated with the aligned reads were subjected to correction and filtering. As a parameter related to cell barcode filtering, the expected number of recovered cells (--expect-cells option) was set to 8000 in this study. The resulting gene-cell UMI count matrices for each sample were then concatenated into one matrix using the “cellranger aggr” pipeline, which also normalized the libraries to the same sequencing depth.

### Data cleaning & normalization

The concatenated gene-cell barcode matrix was imported into Seurat^15^, a toolkit for single-cell RNA-seq data analysis, to undergo data processing. To exclude genes that might be detected from random noise, we filtered genes whose expression was detected in fewer than 3 cells. To exclude poor quality cells that might result from multiplets or other technical noise, we filtered cells that were considered outliers ( > third quartile + 1.5 × interquartile range or < first quartile −1.5 × interquartile range) based on the number of expressed genes detected, the sum of UMI counts and the proportion of UMI counts for mitochondrial genes. Apart from the sequencing depth normalization for different libraries performed during the count matrix concatenation step described above, we normalized the sum of UMI counts for each cell to the median (38,512) of all cells. After normalization, the UMI count data were log-transformed (log (UMI count + 1)).

### Batch effect correction

To correct the potential batch effects in our dataset, we applied a robust approach based on mutual nearest neighbours (MNN)^16^ implemented in the R package scran^17^(the “fastMNN” function; approximate = TRUE, cos.norm = FALSE). For this approach, batch effects not only include systematic differences derived from technical aspects, for example, different library preparation protocols, but also incorporate differences driven by genotypes^16^.

### Data scaling

To mitigate the effects of uninteresting sources of variation, we regressed the effects of mitochondrial gene proportion and UMI count out of the data with linear models implemented in the “ScaleData” function. The data were then centered for each gene by subtracting the average expression of that gene across all cells, after which the data were scaled by dividing the centered expression by the standard deviation.

### Cell cycle effect correction

The cell cycle phase of each cell was inferred based on the expression levels of a panel of phase-specific marker genes (Supplementary Table S2). The “CellCycleScoring” function in the Seurat package was used to assign each cell scores, i.e., G2/M scores and S scores, from which the cell cycle phase could be determined. To remove the cell cycle effect, we regressed out the S scores and G2/M scores during data scaling with the “ScaleData” function. We have made available the scaled expression matrix after the batch and cell cycle effects are removed (gene-cell expression matrix, Data Citation 2).

### Dimensional reduction, clustering & t-SNE projection

Based on expression and dispersion, 1,921 highly variable genes were selected (lower expression cut-off: 0.0125, upper expression cut-off: 3, dispersion cut-off: 0.5). The data on these genes were then subjected to dimensional reduction of the data through principal component analysis. The first 30 principal components were used to cluster the cells into subpopulations through a graph-based unsupervised clustering approach implemented in Seurat (the “FindClusters” function, resolution = 0.4). Following clustering, the same principal components were used to project the clustered cells onto a two-dimensional (2D) map for visualization by means of t-distributed stochastic neighbour embedding (t-SNE). We have make available the clustering information for each cell (cluster_cell_cycle_info_ADSCs, Data Citation 2).

### Code availability

The code for processing the data from a combined raw UMI count matrix to a clean gene-cell matrix is available online (ADSC_scRNAseq_code.R, Data Citation 2).

## Data Records

The sequencing data in the fastq format have been deposited in NCBI Sequence Read Archive (SRA), and are accessible through the project accession number SRP148833 (Data Citation 1). A metadata table (Supplementary Table S1) is available for information such as donor age, sex, liposuction position, experimental manipulation, data generation and deposit for each sample.

## Technical Validation

**Figure 2:**
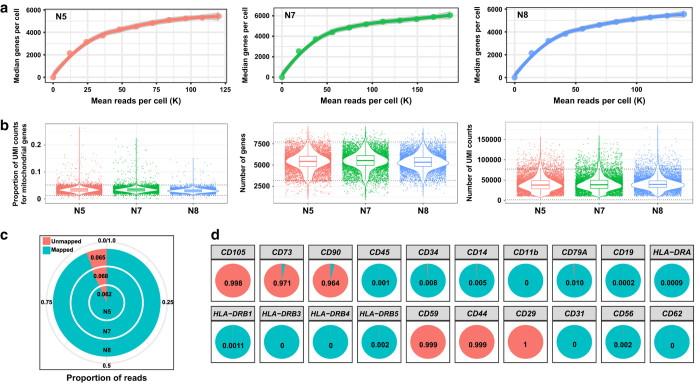
Single-cell RNA-seq data quality assessment and comparison among the three ADSC samples. (**a**) The number of reads sequenced per cell is sufficient for expressed gene detection in each of the three samples. (**b**) The distribution of the proportion of UMI counts for mitochondrial genes, the number of genes detected and the sum of UMI counts in each cell of the three samples. The two dotted lines show the upper and lower threshold used in the cell filtering step. (**c**) The proportion of reads not mapped to the reference genome in the three samples. (**d**) The proportion of cells expressing the ADSC positive and negative marker genes.

We employed the 10x Genomics Chromium platform to construct single cell RNA-seq libraries of three ADSC samples from three healthy donors (Fig. 1a, Supplementary Table S1). Saturation curve analysis indicated that the sequencing depth was almost sufficient for gene detection in each sample, and the median number of genes detected per cell was comparable among the three samples (Fig. 2a). The distribution of the three data quality metrics, i.e., the proportion of UMI counts for mitochondrial genes, the number of genes detected and the sum of UMI counts in each cell were similar among the three samples (Fig. 2b), reflecting little technical variation among samples. To exclude potential low quality data that may result from broken cells, multiplets or other technical issues^18^, we set stringent thresholds for the three data quality metrics, and the high quality data for 24,358 cells were finally retained (Fig. 2b,Table 1). We also noticed that all the three sample could achieve high mapping rates (0.93 on average; Fig. 2c), and the mapping metrics were comparable among the three samples (Supplementary Table S3), reflecting little bias introduced by technical reasons. Taken together, we generated a high quality large-scale single-cell transcriptomic dataset of cultured ADSCs.

For the definition of cultured MSCs, the International Society for Cellular Therapy (ISCT) proposed a minimal criteria for immunophenotypes, i.e., ≥95% of cells expressing markers including CD105, CD73, and CD90, and ≤2% of cells expressing CD45, CD34, CD14 or CD11b, CD19 or CD79A and HLA-DR^19^. We examined the expression of a panel of characteristic marker genes in individual ADSCs. We found that at least 96.4% of the cells expressed the ISCT-proposed positive marker genes *CD105*, *CD73* and *CD90*, and lacked expression of the negative marker genes *CD45*, *CD34*, *CD14*, *CD11b*, *CD19*, *CD79A*, *HLA-DRA*, *HLA-DRB1*, *HLA-DRB3*, *HLA-DRB4* and *HLA-DRB5* (Fig. 2d). Moreover, we examined the expression of some other previously reported human ADSC cell surface marker genes^14^. We found that the expression of the positive marker genes *CD59*, *CD44* and *CD29* could be detected in almost all of the cells, while few cells expressed the negative marker genes *CD31*, *CD56* and *CD62* (Fig. 2d). Taken together, these results suggest that the studied cells constitute a cell population exhibiting characteristic immunophenotypes of cultured ADSCs and with little contamination from other type of cells.

**Figure 3:**
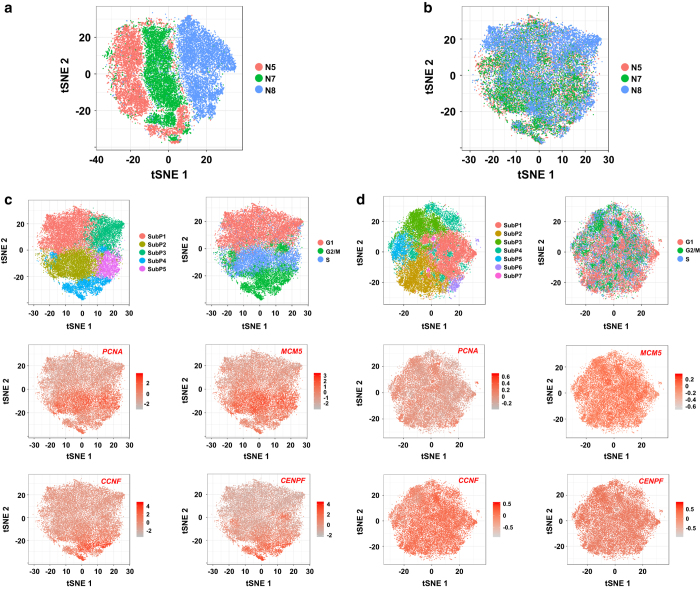
The batch and cell cycle effects are effectively removed from the data. (**a**) The distribution of cells from the three samples in a 2D tSNE space prior to batch effect correction. (**b**) The distribution of cells from the three samples in a 2D tSNE space after batch effect correction. (**c**) Cell cycle represents the dominant source of heterogeneity of the cultured ADSCs when the cell cycle effect is not regressed out of the data. (**d**) The hidden heterogeneity uncovered after the cell cycle effect is regressed out. In c and d, the top left panel displays the subpopulations identified by clustering. The top right panel shows cells labelled by the inferred cell cycle phase (The top right panel). The middle and bottom panels show the expression intensity distribution of the S phase marker genes (*PCNA*, *MCM5*) and G2/M phase genes (*CCNF*, *CENPF*), respectively. Each cell is coloured according to scaled expression of the indicated marker gene.

For single-cell transcriptomic data, potential batch effects must be corrected for datasets from different biological replicates. We noted that, before batch effect correction, the gene expression of cells from the three samples systematically differed from each other, and the three samples were almost separated from each other in a 2D tSNE space, suggesting the presence of batch effects (Fig. 3a). Using a robust MNN-based approach, we successfully removed the batch effects from the data, as reflected by the observation that the cells from the three samples were almost mixed when projected in the 2D tSNE space (Fig. 3b).

In single-cell studies, the effect of cell cycle is often regarded as biologic noise and removed from the data^20^. Initially, we performed clustering when the cell cycle effect was not removed out of the data, and identified five subpopulations at the chosen resolution (Fig. 3c, top left panel). Based on the expression pattern of a panel of marker genes, we scored cell cycle phases for each cell and assigned a cell cycle phase to each cell (Fig. 3c, top right panel). We found that the subpopulations identified by clustering generally correspond to cells inferred to be at the same cell cycle phase: 91.6% of cells in SubP1 were in the G1 phase; 84.7% of cells in SubP3 were also in the G1 phase; 68.8% of cells in SubP2 were at the S phase; 99.6% of cells in SubP4 were identified as G2/M phase cells; and 59.1% of cells in SubP5 were identified as S phase cells. Cells expressing characteristic genes of the same cell cycle phase tended to be clustered together, as exemplified by the expression intensity distribution of the S phase marker genes (*PCNA*, *MCM5*), G2/M phase marker genes (*CCNF*, *CENPF*), which all have peak expression at the specific phases based on the database Cyclebase^21^ (Fig. 3c). These results suggest that cell cycle represents the dominant source of transcriptional heterogeneity in cultured ADSCs, and the hidden heterogeneity may be obscured.

After regressing out the cell cycle effect, by contrast, cells inferred to be at the same cell cycle phase (Fig. 3d, top right panel) were almost mixed in each of the subpopulations (Fig. 3d, top left panel). Cells expressing characteristic genes of the same cell cycle phase were scattered almost randomly throughout the population (Fig. 3d).

Taken together, these results suggest that both the batch and cell cycle effects have been effectively removed from the data, and the resulting clean gene-cell expression matrix would be valuable for further dissecting the intrapopulation heterogeneity at desired clustering resolutions as well as interrogating lineage priming patterns at a single-cell level.

## Additional information

**How to cite this article**: Liu, X. *et al*. Single-cell RNA-seq of cultured human adipose-derived mesenchymal stem cells. *Sci. Data*. 6:190031 https://doi.org/10.1038/sdata.2019.31 (2019).

**Publisher’s note**: Springer Nature remains neutral with regard to jurisdictional claims in published maps and institutional affiliations.

## Supplementary Material



Supplementary Table S1

Supplementary Table S2

Supplementary Table S3

## Figures and Tables

**Table 1:** Summary statistics for the sequencing data of the three ADSC samples.

Sample	Number of cells	Number of Reads	Mean reads per cell	Median genes per cell	Number of cells post filtering
N5	8,906	1,071,156,174	120,273	5,439	7,983
N7	8,478	1,579,342,505	186,287	6,049	7,651
N8	9,256	1,285,218,728	138,852	5,559	8,724
