# Supplementary material for: Single-cell RNA-seq of cultured human adipose-derived mesenchymal stem cells
Source: Sci Data. 2019 Feb 26;6:190031. doi: 10.1038/sdata.2019.31 (PMC6390702; doi:10.1038/sdata.2019.31)
Supplement: Supplementary Table S1 [file sdata201931-s2.pdf]

Table S1. Metadata table for each of the ADSC sample

**SampleID**	N5	N7	N8
**Species**	Homo sapiens	Homo sapiens	Homo sapiens
**Age**	25	28	44
**Sex**	Female	Female	Female
**Weight**	60kg	55kg	51kg
**Liposuction site**	thigh	thigh	thigh
**Protocal1**	Liposuction	Liposuction	Liposuction
**Protocal2**	Cell culture	Cell culture	Cell culture
**Protocal3**	single-cell RNA-seq	single-cell RNA-seq	single-cell RNA-seq
**Library Kit**	10x Genomics Single Cell 3' Reagent Kit	10x Genomics Single Cell 3' Reagent Kit	10x Genomics Single Cell 3' Reagent Kit
**Instrument**	Illumina NovaSeq 6000	Illumina NovaSeq 6000	Illumina NovaSeq 6000
**Layout**	Paired	Paired	Paired
**FlowcellID:laneID**	H3J2VDMXX:1; H3J2VDMXX:2; H3K57DMXX:1; H3K57DMXX:2	H3JWHDMXX:1; H3JWHDMXX:2; H3K3CDMXX:1; H3K3CDMXX:2	H2G3LDSXX:1; H2G3LDSXX:2; H2G3LDSXX:3; H2G3LDSXX:4; H3J55DMXX:1; H3J55DMXX:2
**SRAsampleID**	SRS3334253	SRS3334254	SRS3334255
**Datalink**	https://www.ncbi.nlm.nih.gov/sra/SRX4119295[accn]	https://www.ncbi.nlm.nih.gov/sra/SRX4119294[accn]	https://www.ncbi.nlm.nih.gov/sra/SRX4119296[accn]
