# Supplementary material for: Single-cell RNA-seq of cultured human adipose-derived mesenchymal stem cells
Source: Sci Data. 2019 Feb 26;6:190031. doi: 10.1038/sdata.2019.31 (PMC6390702; doi:10.1038/sdata.2019.31)
Supplement: Supplementary Table S2 [file sdata201931-s3.pdf]

Table S2. Genes that were used to infer the cell cycle phase of each cell

Gene	CellCycleStage
MCM5	S
PCNA	S
TYMS	S
FEN1	S
MCM2	S
MCM4	S
RRM1	S
UNG	S
GINS2	S
MCM6	S
CDCA7	S
DTL	S
PRIM1	S
UHRF1	S
MLF1IP	S
HELLS	S
RFC2	S
RPA2	S
NASP	S
RAD51AP1	S
GMNN	S
WDR76	S
SLBP	S
CCNE2	S
UBR7	S
POLD3	S
MSH2	S
ATAD2	S
RAD51	S
RRM2	S
CDC45	S
CDC6	S
EXO1	S
TIPIN	S
DSCC1	S
BLM	S
CASP8AP2	S
USP1	S
CLSPN	S
POLA1	S
CHAF1B	S
BRIP1	S
E2F8	G2M
HMGB2	G2M
CDK1	G2M
NUSAP1	G2M
UBE2C	G2M
BIRC5	G2M
TPX2	G2M
TOP2A	G2M
NDC80	G2M
CKS2	G2M
NUF2	G2M
CKS1B	G2M
MKI67	G2M
TMPO	G2M
CENPF	G2M

TACC3	G2M
FAM64A	G2M
SMC4	G2M
CCNB2	G2M
CKAP2L	G2M
CKAP2	G2M
AURKB	G2M
BUB1	G2M
KIF11	G2M
ANP32E	G2M
TUBB4B	G2M
GTSE1	G2M
KIF20B	G2M
HJURP	G2M
CDCA3	G2M
HN1	G2M
CDC20	G2M
TTK	G2M
CDC25C	G2M
KIF2C	G2M
RANGAP1	G2M
NCAPD2	G2M
DLGAP5	G2M
CDCA2	G2M
CDCA8	G2M
ECT2	G2M
KIF23	G2M
HMMR	G2M
AURKA	G2M
PSRC1	G2M
ANLN	G2M
LBR	G2M
CKAP5	G2M
CENPE	G2M
CTCF	G2M
NEK2	G2M
G2E3	G2M
GAS2L3	G2M
CBX5	G2M
CENPA	G2M
