# Supplementary material for: Single-cell RNA-seq of cultured human adipose-derived mesenchymal stem cells
Source: Sci Data. 2019 Feb 26;6:190031. doi: 10.1038/sdata.2019.31 (PMC6390702; doi:10.1038/sdata.2019.31)
Supplement: Supplementary Table S3 [file sdata201931-s4.pdf]

Table S3. The mapping metrics for the three ADSC samples

Sample	Reads Mapped to Genome	Reads Mapped Confidently to Genome	Reads Mapped Confidently to Intergenic Regions	Reads Mapped Confidently to Intronic Regions	Reads Mapped Confidently to Exonic Regions	Reads Mapped Confidently to Transcriptome	Reads Mapped Antisense to Gene
N5	93.50%	91.00%	2.50%	12.90%	75.70%	72.00%	1.20%
N7	93.20%	90.60%	2.60%	12.80%	75.20%	71.50%	1.10%
N8	93.80%	91.30%	2.60%	12.50%	76.20%	72.70%	1.00%
